# Microbial diversity and structure in the gastrointestinal tracts of two stranded short‐finned pilot whales (*Globicephala macrorhynchus*) and a pygmy sperm whale (*Kogia breviceps*)

**DOI:** 10.1111/1749-4877.12502

**Published:** 2020-12-02

**Authors:** Shijie BAI, Peijun ZHANG, Mingli LIN, Wenzhi LIN, Zixin YANG, Songhai LI

**Affiliations:** ^1^ Institute of Deep‐sea Science and Engineering Chinese Academy of Sciences Sanya China

**Keywords:** gastrointestinal tract, gut microbiome, microbial diversity, pygmy sperm whale (*Kogia breviceps*), short‐finned pilot whale (*Globicephala macrorhynchus*)

## Abstract

Information on the gut microbiome composition of different mammals could provide novel insights into the evolution of mammals and succession of microbial communities in different hosts. However, there is limited information on the gut microbiome composition of marine mammals, especially cetaceans because of sampling constraints. In this study, we investigated the diversity and composition of microbial communities in the stomach, midgut, and hindgut of 2 stranded short‐finned pilot whales (*Globicephala macrorhynchus*) and hindgut of a stranded pygmy sperm whale (*Kogia breviceps*) by using 16S rRNA gene amplicon sequencing technology. On the basis of the 50 most abundant operational taxonomic units, principal coordinate analysis, and non‐metric multidimensional scaling analysis, we confirmed that the gut microbial communities of the 3 whales were different. Our results revealed that the gut microbiome of 1 stranded short‐finned pilot whale GM16 was dominated by Firmicutes (mainly *Clostridium*) and Fusobacteria; whereas that of the other pilot whale GM19 was composed of Gammaproteobacteria and Bacteroidetes (mainly *Vibrio* and *Bacteroides*, respectively), probably caused by intestinal disease and antibiotic treatment. The gut microbiome of the pygmy sperm whale was dominated by Firmicutes and Bacteroidetes. Moreover, different gastrointestinal tract regions harbored different microbial community structures. To our knowledge, this is the first report of the gut microbiome of short‐finned pilot whales, and our findings will expand our current knowledge on microbial diversity and composition in the gastrointestinal tract of cetaceans.

## Graphical abstract

Gut microbial communities of the pilot and sperm whales are different, and different gastrointestinal tract regions harbored different microbial community structures. Firmicutes and Fusobacteria are the dominant gut microbes in pilot whales, whereas Firmicutes and Bacteroidetes are the dominant gut microbes in pygmy sperm whale.

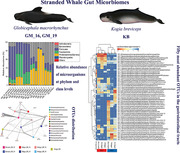

## INTRODUCTION

Microorganisms are extremely abundant and diverse in the gut of mammals (Gensollen *et al*. 2016). The interaction between microorganisms and their host cells is necessary for the health, survival, and regulation of physiological functions of the host (Krajmalnik‐Brown *et al*. 2012; Woting & Blaut 2016; Dang & Marsland 2019). These microorganisms and their associated phenomes shape the host immune system and contribute to nutrient absorption and defense against infectious diseases in the host (Dzutsev *et al*. 2017; Quin & Gibson 2020). There are many studies focusing on the microbiomes in the gastrointestinal tracts of some terrestrial mammals, such as the giant panda (*Ailuropoda melanoleuca*) (Xue *et al*. 2015) and the cattle (Li *et al*. 2019). Moreover, the gut microbiomes in humans have been studied extensively (McKenney & Pamer 2015; Dzutsev *et al*. 2017; Quin & Gibson 2020). Studies on the microbial diversity and composition in the gastrointestinal tracts of cetaceans are very few. To our knowledge, only 3 papers investigated the microbial communities in different regions of the gastrointestinal tracts in cetaceans (Wan *et al*. 2018, 2020; Tian *et al*. 2020).

The diversity, structure, and function of the mammalian gut microbiome were reported to be mainly shaped by diet adaptation (Ley *et al*. 2008a,b; Muegge *et al*. 2011). Cetaceans have been learned to evolve from herbivorous terrestrial artiodactyls related to cows and hippopotamuses (Gatesy *et al*. 2013). Nevertheless, their diets obviously differ in that cetaceans feed exclusively on animals, while herbivorous terrestrial artiodactyls on only grasses. The microbial diversity and functional potential of the gut microbiomes of baleen whales were compared with those of other terrestrial mammals, and the baleen whales were found to harbor unique gut microbiomes with a functional capacity similar to that of both carnivores and herbivores (Sanders *et al*. 2015). Some researchers have indicated that host habitat, diet, and phylogeny all contribute to variations in the gut microbial composition of marine mammals (Bik *et al*. 2016). Recently, high‐throughput sequencing technology has facilitated the study of gut microbiomes in different marine mammals, such as seals (Pacheco‐Sandoval *et al*. 2019), sea lions (Lavery *et al*. 2012), and manatees (Merson *et al*. 2014). However, challenges in the sampling of some marine mammals, especially cetaceans, have resulted in limited information on gut microbiome diversity. A few studies have been conducted, for example, studies based on comparison of fecal samples from whales, including the blue whale (*Balaenoptera musculus*) (Guass *et al*. 2016), several other dolphins and whales (Sanders *et al*. 2015), and the pygmy sperm whale (*Kogia breviceps*) and dwarf sperm whale (*K. sima*) (Erwin *et al*. 2017). However, knowledge on the composition of their gut microbiomes is limited, leading to poor understanding of the gut microbiomes of marine mammals, for example, composition divergence and convergence, composition shaping factors, and species‐specific ranges.

The short‐finned pilot whale (*Globicephala macrorhynchus*) is a globally distributed offshore and deep‐diving odontocete cetacean that belongs to the family Delphinidae. Short‐finned pilot whales inhabit warm temperate waters, such as tropical and subtropical waters (Jefferson*et al*. 2015). They feed on squid and fishes such as cod, turbot, mackerel, hake, and spiny dogfish (Olson 2009). The pygmy sperm whale is one of 3 species in the sperm whale family, and it is a deep‐diving species. Analysis of the stomach contents of pygmy sperm whales suggest that they feed primarily on cephalopods such as glass squid, lycoteuthid and ommastrephid squid, octopus, and deep‐sea shrimp (Bloodworth & Odell 2008). Here, we investigated the microbiomes in the gastrointestinal tract samples, including stomach, midgut, and hindgut samples, from 2 short‐finned pilot whales and a hindgut sample from a pygmy sperm whale. We aimed to (1) provide insights into the gut microbial composition and community structure in different parts of the gastrointestinal tract of two deep‐diving cetacean species and (2) collect basic data for understanding variation patterns in the gut microbial composition of marine mammals.

## MATERIAL AND METHODS

### Sample collection

**Figure 1:**
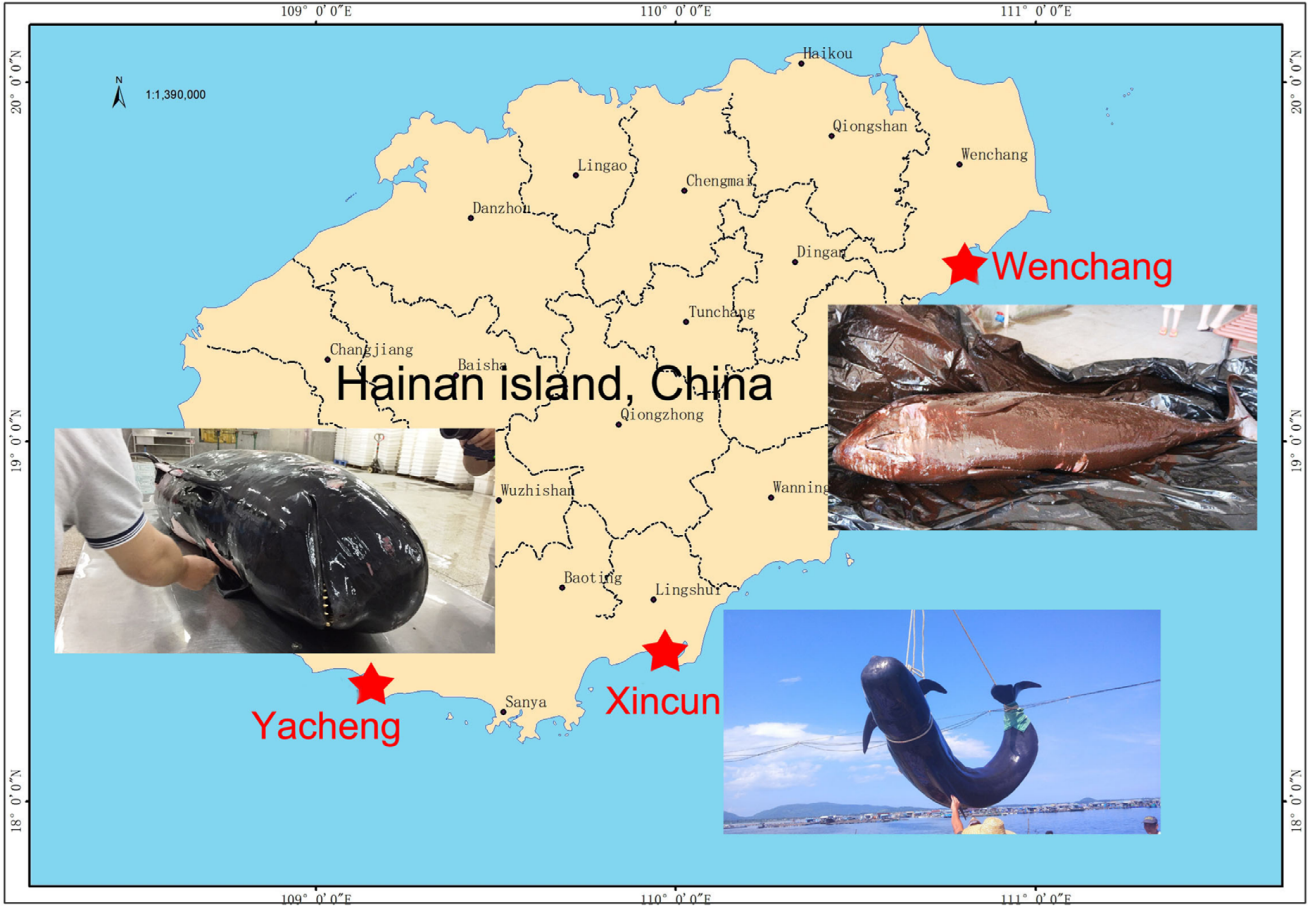
Locations where the 3 cetaceans investigated in this study were stranded.

Gastrointestinal tract samples, including stomach, midgut, and hindgut samples, from 2 stranded short‐finned pilot whales and 1 hindgut sample from a stranded pygmy sperm whale were collected at the coast of Hainan Island, China. On July 8, 2016, the first short‐finned pilot whale (assigned as GM16) was found dead (fresh carcass, code 2; Geraci & Lounsbury 2005) in the coastal waters of Xincun, Lingshui, Hainan Island, China. On the same day, the dead whale was stored immediately after sample collection in a refrigeration house at −20 °C and frozen for 20 days before necropsy. It was an adult female, with a body length of 3.68 m and body mass of 550 kg. On June 7, 2019, the second short‐finned pilot whale (assigned as GM19) was found stranded in the waters of Yacheng, Sanya, Hainan Island, China, and transferred to a rescue center with a net pen in open water, after medical examination, intravenous ceftriaxone sodium was used 10 g per day in 2 days for treatment. The whale died on June 10, 2019, about 3 days after the stranding, and was stored in a refrigeration house at 4 °C about 6 h after death and necropsied on the next day. It was an adult female with a body length of 2.98 m and body mass of 343 kg. To avoid species misrecognition, the 2 pilot whales aforementioned were also verified through mtDNA sequencing. On June 15, 2014, a pygmy sperm whale (assigned as KB) was found stranded in the coastal waters of Wenchang, Hainan Island, China, and transferred to a rescue center with a concrete pool for treatment on the same day. This whale died 3 days later, and it was stored at a refrigeration house at −20 °C about 4 h after death. It was frozen for 37 days before necropsy, and it was an adult female with a body length of 2.7 m and body mass of 280 kg. The locations where the three cetaceans were stranded are shown in Fig. 1. The gastrointestinal tract samples were collected during the necropsies. For each sampled whale, the stomach samples were taken from the main stomach, the midgut samples were from the middle of the intestinal tract, and the hindgut samples were from the colon and rectum. Each sample was the content of the intestinal tract with a length of approximately 100 to 120 cm long, except the hindgut sample of KB, which was just the content from around 25 cm long rectum section. The content of each pilot whale sample was analyzed in triplicate whereas the hindgut sample of KB was analyzed only once due to sample limitation. All samples were stored at −80 °C for DNA extraction.

### DNA extraction and sequencing

DNA was extracted from the gut samples (2 blank control samples were used) with MoBio PowerSoil extraction kits (Mo Bio Laboratories, Carlsbad, CA, USA), according to the manufacturer's instructions. The extracted DNA was quantified with a Qubit fluorometer (Invitrogen Inc. Manufacturer: Life Technologies Holdings Pte Ltd, Singapore). Bacterial and archaeal 16S rRNA were amplified with the primer set 515f Modified and 806r Modified (Walters *et al*. 2015). The PCR cycling conditions were followed the method previously described (Bai & Hou 2020). The PCR products were purified with TaKaRa purification kit (TaKaRa, Japan). The PCR products were prepared for constructing the 16S rRNA gene libraries with the TruSeq DNA sample preparation kit (Illumina, San Diego, CA, USA), according to the manufacturer's instructions. The Illumina sequencing was performed with the MiSeq platform (Illumina) and run at MajorBio Co. Ltd. (Shanghai, China).

### Microbial community analysis

After sequencing, the raw reads were split to samples according to the different sample barcodes, and the forward and reverse primers of all raw reads were trimmed. One mismatch of these 2 processing steps was allowed. Overlapping clean reads were merged using FLASH (Magoč & Salzberg 2011) with at least a 30 bp overlap into full‐length sequences. The threshold, including a quality score >20 and window size of 5, was used to remove the low quality sequences via the Btrim program (Kong 2011), and any sequences containing N's or ambiguous bases were discarded. Only sequences approximately 253 bp in length were treated as targeted sequences. Operational taxonomic units (OTUs) were generated based on 97% cutoff of sequences similarity, and the longest sequence of each OTU were used as the representative sequences. Meanwhile, the chimeras were discarded, all these processing were conducted by UPARSE (Edgar 2013); the singletons were retained for further analysis. The representative sequence of each OTU was selected for taxonomic annotation by comparison with the SILVA 128 database (Quast *et al*. 2013). The OTUs were randomly resampled to normalize the reads of each sample. The raw sequencing reads obtained from the Illumina sequencing of 16S rRNA genes were deposited in the NCBI database (http://www.ncbi.nlm.nih.gov/) under BioProject accession number PRJNA631404.

### Statistical analysis

The microbial diversity of the communities from the gastrointestinal tracts of GM16 and GM19 were determined by statistical analysis of the α‐diversity indices. The Shannon and Inverse Simpson indices were calculated by the vegan package in R language (R Core Team 2018). The Chao1 values (Chao 1984) were generated using the Mothur program (Schloss *et al*. 2009). The chosen representative OTUs of all samples were aligned by PyNAST (Caporaso *et al*. 2010). The tree file was generated from FastTree (Price *et al*. 2009), subsequent processing then to calculate the phylogenetic diversity with the Picante package in R (Kembel *et al*. 2010). The random forest analysis was also conducted in R using the randomForest package, and ß‐diversity‐based statistical tools, such as principal coordinate analysis (PCoA) on the basis of weighted UniFrac distance and non‐metric multidimensional scaling (NMDS) with Bray–Curtis distance matrix, were used to test the differences within the microbial community structure. The detailed analyses were described previously (Bai & Hou 2020). Data comparison between different groups was performed by the *Wilcoxon rank‐sum* test using IBM SPSS Statistics 19.

### Phylogenetic analysis

Each target OTU nucleotide sequence was uploaded in the NCBI website by using BLASTn against the 16S ribosomal RNA sequence database. The nucleotide sequences of 19 closest relatives and 1 far relative of each target OTU were selected and aligned using MAFFT v7.397 (Katoh & Standley 2013) with the nucleotide sequence of the target OTU. Phylogenetic trees were constructed using the maximum‐likelihood method. The topology of the phylogenetic trees was evaluated using bootstrap resampling with 1000 replicates in MEGA7 (Kumar *et al*. 2016).

## RESULTS

### Sequencing statistics and microbial diversity

**Figure 2:**
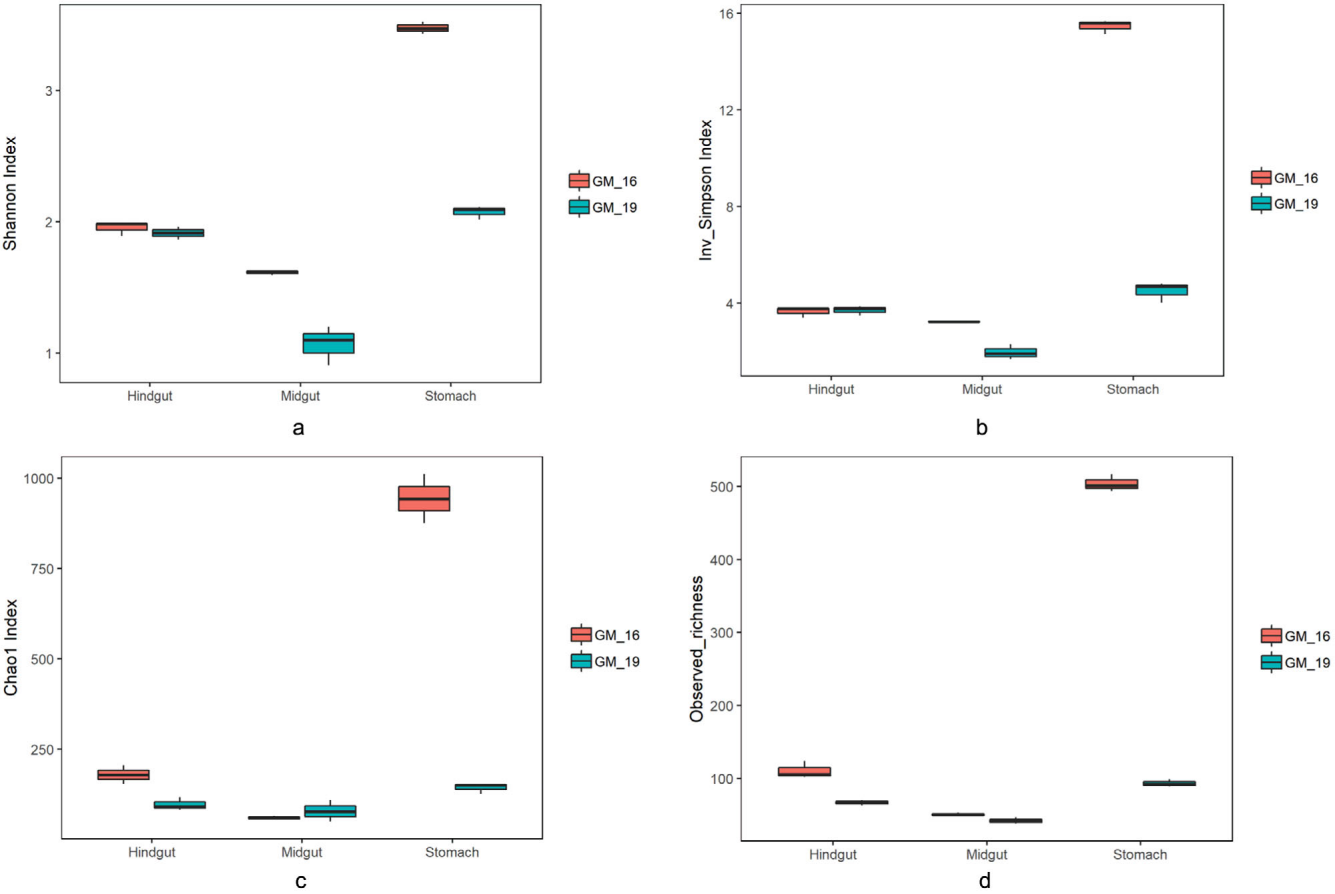
Comparisons of 4 α‐diversity indices, Shannon index (a), inverse Simpson index (b), Chao1 index (c), and observed richness (d), of the short‐finned pilot whales. GM16 refers to *Globicephala macrorhynchus* stranded in 2016; GM19 refers to *G. macrorhynchus* stranded in 2019.

A total of 1 230 195 sequences were obtained from 19 gut samples after quality assessment. An average of 64 747 ± 6065 sequences were obtained from each sample. In order to get more accurate result of α‐diversity, we randomly resampled 51 431 sequences of each sample, and then applied for the next analyses of microbial diversity, composition, and structure. The α‐diversities of microbial communities from the gastrointestinal tracts of GM16 and GM19 were calculated. The Shannon, Inverse Simpson, and Chao1 indices and observed richness all indicated that the α‐diversity of the gut microbiome from GM16 was higher than that from GM19 (Fig. 2). We also found that the α‐diversity was lower in the midgut than in the hindgut in both GM16 and GM19; however, no statistical difference was detected (*Wilcoxon rank‐sum* test, *P* > 0.05).

### Structure and composition of the microbial communities

**Figure 3:**
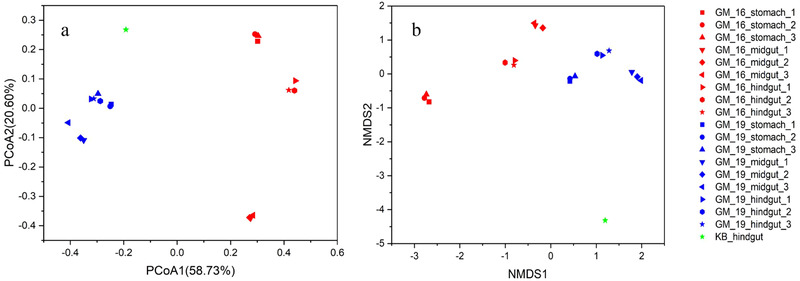
Principal coordinate analysis (PCoA) on the basis of weighted UniFrac distance (a), and non‐metric multidimensional scaling analysis (NMDS) with Bray–Curtis distance matrix (b) to visualize the structure of microbial communities in the stomach, midgut, and hindgut of GM16 and GM19, and hindgut of KB of the detected OTUs. GM16 refers to *Globicephala macrorhynchus* stranded in 2016; GM19 refers to *G. macrorhynchus* stranded in 2019; KB refers to *Kogia breviceps* stranded in 2014.

We successfully sequenced the samples from the stomach, midgut, and hindgut of both GM16 and GM19 and one sample from the hindgut of KB. PCoA and NMDS analysis of microbial communities clearly separated these 3 cetacean samples (Fig. 3), suggesting that the 3 individuals harbored different gut microbial communities. Furthermore, the multi‐response permutation procedure (MRPP), one‐way ordered analysis of similarity (ANOSIM), and permutational multivariate analysis of variance (PERMANOVA) showed significant differences between GM16 and GM19 (Table 1).

**Table 1:** Dissimilarity analyses of gut microbial communities in 2 stranded short‐finned pilot whales on the basis of Jaccard and Bray–Curtis distance

	Jaccard		Bray–Curtis	
PERMANOVA	*F*	*P*	*F*	*P*
Gut microbiome (GM16 & GM19)	5.6698	0.002(**)	13.1845	0.001(***)
Gut microbiome (GM16 & GM19)	Jaccard		Bray–Curtis	
	*P*		*P*	
MRPP	0.001(***)		0.001(***)	
ANOSIM	0.001(***)		0.001(***)	

* Difference is significant at 0.05 level; ** difference is significant at 0.01 level; *** difference is significant at 0.001 level. GM16 refers to *Globicephala macrorhynchus* stranded in 2016, GM19 refers to *G. macrorhynchus* stranded in 2019.

**Figure 4:**
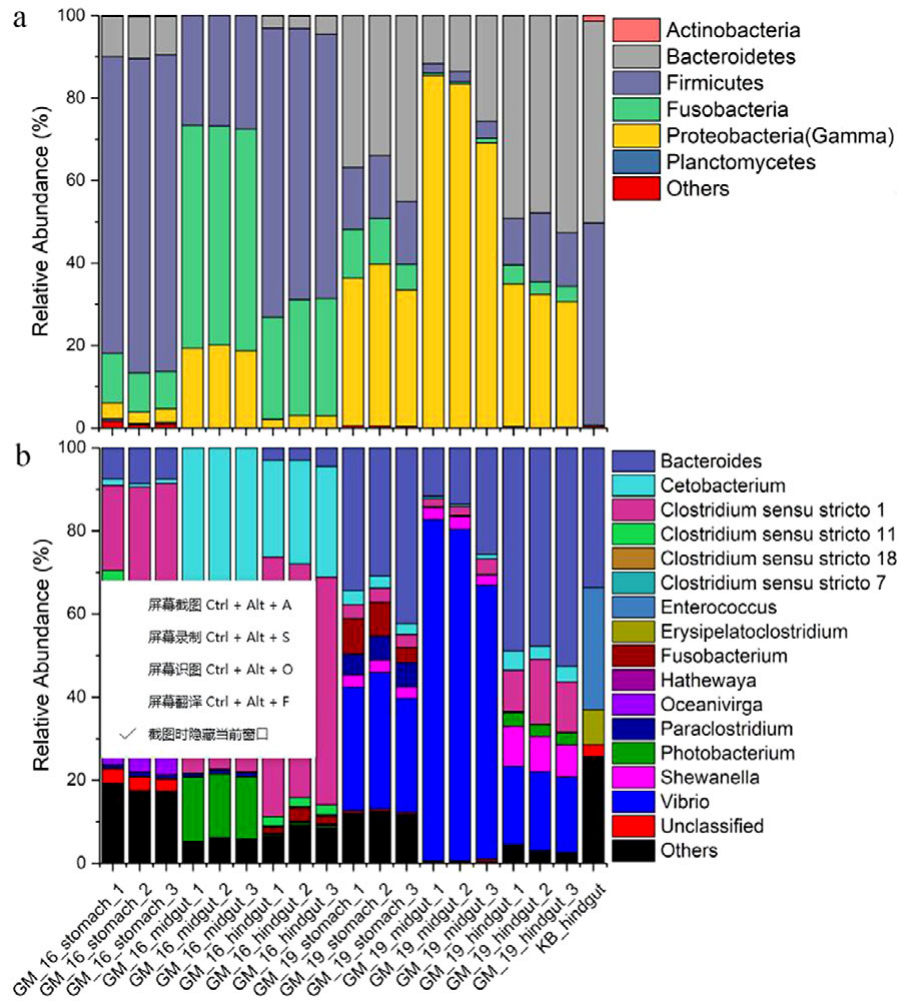
Stacked bar chart showing relative abundance of microorganisms in the stomach, midgut, and hindgut of GM16 and GM19, and hindgut of KB at the phylum and class levels (a), and genus level (b). GM16 refers to *Globicephala macrorhynchus* stranded in 2016; GM19 refers to *G. macrorhynchus* stranded in 2019; KB refers to *Kogia breviceps* stranded in 2014.

**Figure 5:**
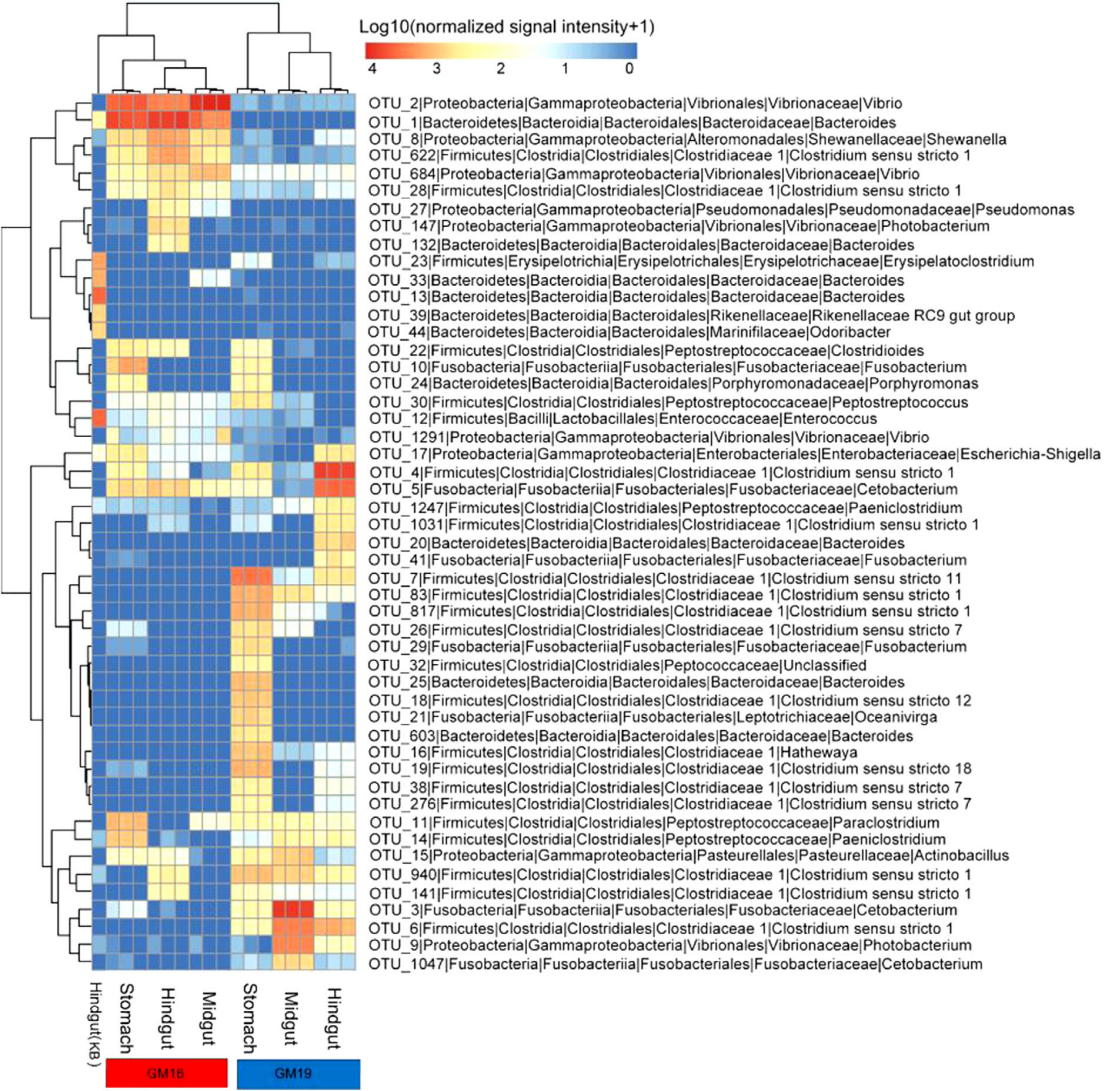
Fifty most abundant OTUs in the gastrointestinal tracts of GM16 and GM19, and hindgut of KB. Microbial abundance was scaled with log transformation in the heatmap. GM16 refers to *Globicephala macrorhynchus* stranded in 2016; GM19 refers to *G. macrorhynchus* stranded in 2018; KB refers to *Kogia breviceps* stranded in 2014.

The relative abundance of microorganisms was evident at phylum, class, and genus levels, with a similarity of 97% for OTU classification, and provided detailed information on the composition of the microbial communities (Fig. 4). The dominant microbial phyla were Firmicutes and Fusobacteria, which comprised 26.57% to 76.72% and 9.07% to 54.08% of all gut samples in GM16, respectively. Gammaproteobacteria (30.52% to 85.43%) and Bacteroidetes (11.68% to 49.16%) were dominant in the gut samples of GM19. Firmicutes (49.04%) and Bacteroidetes (48.91%) were the dominant phyla in the hindgut of KB (Fig. 4). Furthermore, the genus level was dominated by *Cetobacterium* (0.83% to 54.08%) and *Clostridium sensu stricto*, including *Clostridium sensu stricto* 1, 7, 11, and 18, and they accounted for 24.41% and 65.05% of the sequences, respectively, in the gut samples of GM16. In the gut samples of GM19, *Vibrio* (18.14% to 82.26%) and *Bacteroides* (11.68% to 52.56%) were dominant (Fig. 4). The relative abundances of *Vibrio* and *Bacteroides* were significantly higher (*Wilcoxon rank‐sum* test, *P* < 0.01) in the gastrointestinal tract of GM19 than in the gastrointestinal tract of GM16, whereas the relative abundance of *Clostridium sensu stricto* was significantly lower (*Wilcoxon rank‐sum* test, *P* < 0.01) in the gastrointestinal tract of GM19 than in that of GM16. The relative abundance of *Cetobacterium* was also lower in the gastrointestinal tract of GM19 than in that of GM16, but no statistical difference was detected (*Wilcoxon rank‐sum* test, *P* > 0.05). The 50 most abundant OTUs were analyzed using the randomForest package in R. The results showed that the overall out‐of‐bag error was 0%; the class error of GM16 and GM19 was also 0%, which suggests that the 50 most abundant OTUs could represent the vast majority of microbial communities in the gastrointestinal tracts of GM16, GM19, and KB. The heatmap based on the 50 most abundant OTUs indicated that GM16 and GM19 harbored distinct microbial communities and differed from those in the hindgut of KB (Fig. 5).

### Phylogenetic analysis of key OTUs

**Figure 6:**
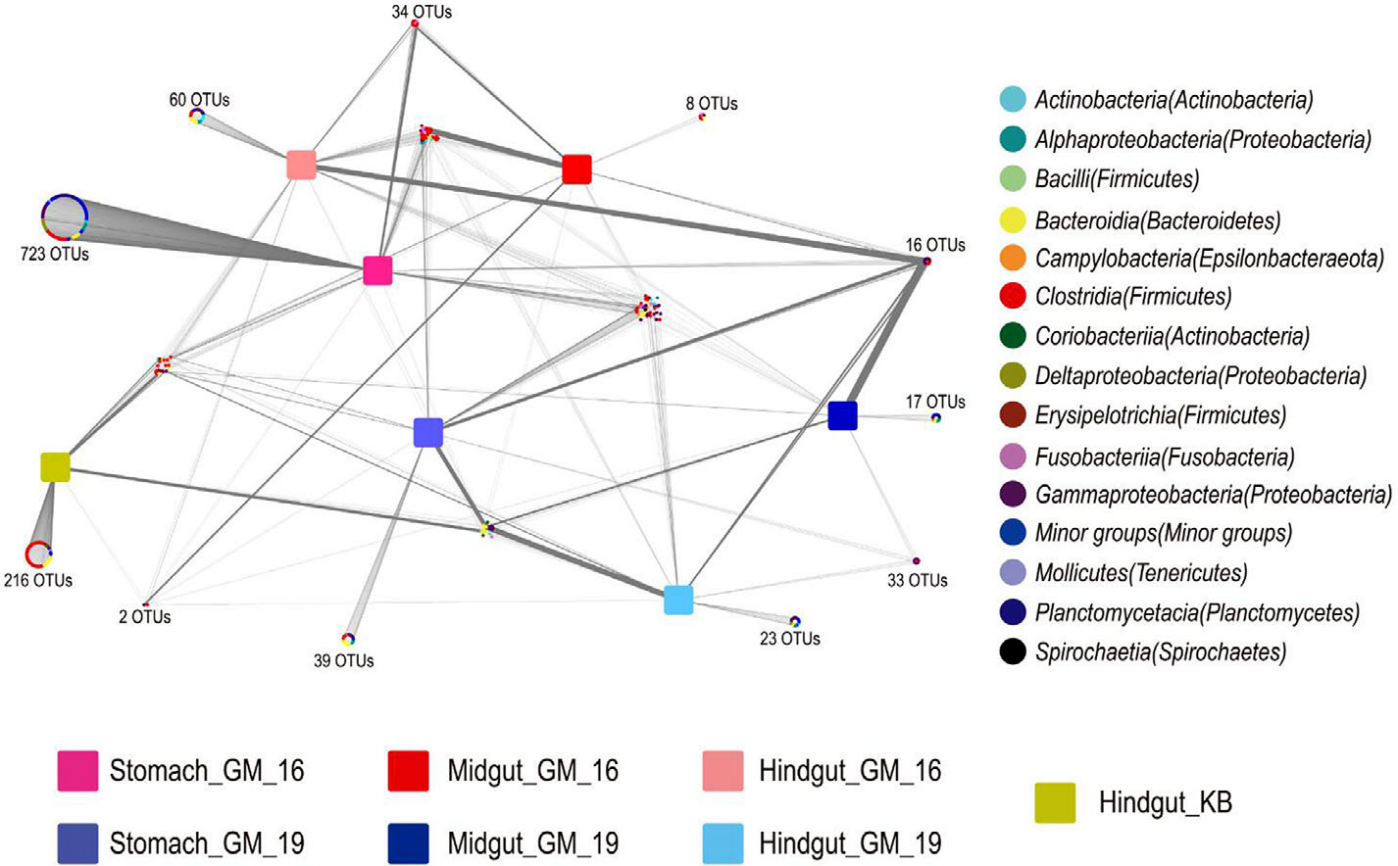
Distribution of OTUs in the stomach, midgut, and hindgut of GM16 and GM19, and hindgut of KB. GM16 refers to *Globicephala macrorhynchus* stranded in 2016; GM19 refers to *G. macrorhynchus* stranded in 2019; KB refers to *Kogia breviceps* stranded in 2014.

Quality control and random resampling of the 19 samples were conducted, and the sequence reads were clustered into 1261 OTUs at 97% similarity level. In GM16, 723, 8, and 60 unique OTUs were found in the stomach, midgut, and hindgut, respectively; in GM19, 39, 17, and 23 unique OTUs were detected in the stomach, midgut, and hindgut, respectively. Besides, 216 unique OTUs were found in the hindgut of KB. Sixteen OTUs were present in all gut samples of GM16 and GM19. However, only 2 OTUs were shared between the samples of the short‐finned pilot whales and hindgut sample of KB (Fig. 6). Furthermore, OTU1 and OTU2 were the most abundant OTUs in the gut samples of GM19 and comprised 33.54% and 37.38% sequence reads of GM19, respectively. The phylogenetic analysis based on the maximum‐likelihood method showed that the strains *Bacteroides fragilis* ATCC 25285 and *Vibrio cidicii* 2756–81 were most closely related to OTU1 and OTU2, respectively (Fig. S1a,b, Supporting Information). OTU9 and OTU1247 were present in the samples of the short‐finned pilot whales and hindgut sample of KB. The phylogenetic analysis showed that OTU9 was closely related to *Photobacterium damselae*, and OTU1247 was closely related to *Paeniclostridium sordellii* (Fig. S1c,d, Supporting Information). The 16 OTUs shared between the gut samples of GM16 and GM19 were analyzed, and the results showed that most of the shared OTUs (56.3%) were related to *Vibrio* and *Clostridium sensu stricto 1*. The rest of the OTUs were assigned to *Actinobacillus*, *Cetobacterium*, *Enterococcus*, *Escherichia‐Shigella*, *Paeniclostridium*, *Shewanella*, and *Vagococcus*.

At last, we want to point out that, we detected many opportunistic and pathogenic strains in GM19, such as *V. cidicii* (37.38%) and *B. fragilis* (33.54%) comprising the most abundant OTUs, which can cause inflammatory bowel diseases indicating that this animal suffered from serious enteritis and could be a possible reason for the death of GM19, although no definitive pathology test verified this with 100% accuracy.

## DISCUSSION

### Diversity of the gut microbiomes in the short‐finned pilot whales

In the present study, samples were obtained from the stomach, midgut, and hindgut of 2 stranded short‐finned pilot whales at different sites and in different years, and 1 hindgut sample was collected from a stranded pygmy sperm whale. The α‐diversity indices and observed richness showed that the gut microbial diversity of GM16 was higher than that of GM19. The reason for the differences in α‐diversity is complicated and could be attributed to the different diets of the animals and health conditions; for example, they may be suffering from a disease or treated with antibiotics during the rescue. A previous study showed that the α‐diversity of fecal microbial communities is similar in sister species or same species, for example, between pygmy sperm whales and dwarf sperm whales (*K. sima*) (Erwin *et al*. 2017) and between two adult blue whales (Guass *et al*. 2016).

We cannot exactly explain why the gut microbial diversity of GM16 was higher than that of GM19. We suppose that antibiotics treatment and potential intestinal disease in GM19 might be the most probable reasons. Alternatively, the location‐ and sampling year‐mediated differences such as diet and living environment could also contribute to intestinal microbial dissimilarities between individuals. Further studies are warranted. The α‐diversity of the stomach microbial communities was observed to be higher than those of the midgut and hindgut communities, while the midgut harboring a lower but not significant microbial diversity than in the hindgut (*Wilcoxon rank‐sum* test, *P* > 0.05). Previous limited studies showed that the stomach harbored high microbial diversity, and for the intestinal tract, the posterior compartment could be more suitable for microbial colonization across cetaceans and other animal species (e.g. Wan *et al*. 2018; Tian *et al*. 2020). Our results are consistent with those views.

### Microbial community structure and composition in the gastrointestinal tracts of the short‐finned pilot whales and pygmy sperm whale

The results of PCoA, NMDS, and dissimilarity tests revealed that the gut microbial community structures differed significantly in the 2 stranded short‐finned pilot whales, and the microbial community structures of the stomach, midgut, and hindgut were different. In a previous study of microbial communities along the gastrointestinal tract of East Asian finless porpoises, the bacterial community structures in the stomach differed from those in the foregut, whereas the bacterial community structures of the foregut, hindgut, and feces could not be separated using NMDS analysis (Wan *et al*. 2018). This may be due to species or statistical differences. However, further studies on more individuals are needed to evaluate this issue.

Members of Firmicutes, Gammaproteobacteria, Bacteroidetes, and Fusobacteria constituted the vast majority of the gut microorganisms in the 2 short‐finned pilot whales and pygmy sperm whale. However, the distribution of gut microorganisms was different in the 2 short‐finned pilot whales. For instance, Firmicutes and Fusobacteria were dominant in the gastrointestinal tract of GM16, whereas Gammaproteobacteria and Bacteroidetes were dominant in that of GM19. In the hindgut sample of the pygmy sperm whale, most of the microorganisms belonged to Firmicutes and Bacteroidetes, which is consistent with the findings of previous studies on the fecal microbial composition of stranded sperm whales (*Physeter catodon*), pygmy sperm whales, and dwarf sperm whales (Erwin *et al*. 2017; Li *et al*. 2019). Furthermore, Actinobacteria members can be found in the gut microbiomes of kogiids.

Members of the genus *Clostridium* (phylum, Firmicutes) have been identified as the main lineages of gut microbiomes in blue whales, and very few reads can be assigned to the phylum Proteobacteria (Guass *et al*. 2016). Similarly, in the gut microbiomes of kogiids, the lineages of Proteobacteria have been classified as minor groups (Erwin *et al*. 2017), and the same results were obtained from the gut microbiomes of humpback whales (*Megaptera novaeangliae*), North Atlantic right whales (*Eubalaena glacialis*), and sei whales (*Balaenoptera borealis*). In contrast, a relatively high abundance of members that belong to Proteobacteria have been found in the fecal samples of common bottlenose dolphins (*Tursiops truncatus*), East Asian finless porpoises, and belugas (*Delphinapterus leucas*) (Sanders *et al*. 2015; Erwin *et al*. 2017; Wan *et al*. 2018). In general, microorganisms that belong to Proteobacteria may be related to the food of their hosts, zooplankton predators (i.e. baleen whales) and cephalopod predators (i.e. short‐finned pilot whales and kogiids) are different from piscivorous predators such as common bottlenose dolphins, East Asian finless porpoises, and belugas. However, it is very rare to find members of the genus *Vibrio* in the gastrointestinal tracts of toothed whales. In our study, the lineages of *Vibrio* were dominant in the stomach (≈30.05%), midgut (≈76.01%), and hindgut (≈18.65%) of GM19. However, we detected very limited *Vibrio* in the gastrointestinal tract of GM16 (≈0.01%) and no *Vibrio* in the hindgut sample of KB. In addition, OTU2, which comprises 37.38% sequences of GM19, was closely related to *V. cidicii*. Isolates of *V. cidicii* have been obtained from human clinical blood samples by the Centers for Disease Control and Prevention (CDC, Atlanta, GA, USA); it is the closest known relative of *Vibrio navarrensis* and can be a potentially opportunistic pathogen that is highly lethal to humans who consume shrimp, fish, oysters, and clams (Jones & Oliver 2009; Orata *et al*. 2016). Another dominant OTU, which comprises 33.54% sequences of GM19, was closely related to *B. fragilis*, which is an enterotoxigenic bacterium that can cause inflammatory bowel diseases and colorectal cancer (Rhee *et al*. 2009). The vast number of *Vibrio* and *Bacteroides* members detected in the gastrointestinal tract of GM19 indicated that this animal suffered from serious enteritis. Therefore, enteritis due to pathogenic *Vibrio* and *Bacteroides* in the gastrointestinal tract were supposed to be a possible cause for the death of GM19. Also, we suppose that intestinal disease and antibiotic treatment could be the most probably reasons that caused different dominant microbial phyla between GM16 and GM19.

Members of *Clostridium* are dominant and widely distributed in certain cetacean hosts, such as blue whales (Guass *et al*. 2016), sperm whales (Li *et al*. 2019), pygmy sperm whales, and dwarf sperm whales (Erwin *et al*. 2017). In the gastrointestinal tract of GM16, a large number of *Clostridium* members were detected, especially in the stomach and hindgut. Moreover, several putative pathogens, such as *Clostridium perfringens* and *Clostridium botulinum*, were found in the gut samples of GM16, and these opportunistic and pathogenic microorganisms may often be responsible for the stranding and death of cetaceans (Waltzek *et al*. 2012). *Clostridium* is one of the most common genus found among the gut microorganisms of cetaceans, suggesting that *Clostridium* members have low virulence and can be a potential threat to unhealthy cetaceans (Erwin *et al*. 2017; Marón *et al*. 2019). Members that belong to the genus *Cetobacterium* from the phylum Fusobacteria were also detected in the gastrointestinal tracts of both GM16 and GM19, but not in the hindgut sample of KB. *Cetobacterium* has been previously identified in the gut of toothed whales (Sanders *et al*. 2015) and some other marine mammals such as Antarctic seals and southern right whales (*Eubalaena australis*) (Nelson *et al*. 2013a; Marón *et al*. 2019). The relative abundance of the phylum Fusobacteria is significantly higher in the gut microbiome of marine mammals than in that of terrestrial mammals; the percentage of microorganisms from the phylum Fusobacteria is significantly higher in marine carnivores than in the other dietary marine mammal groups (Nelson *et al*. 2013b).

The findings of the present study expand our knowledge on the gut microbiome of cetaceans. However, we have to mention that all the 3 investigated whales were deep‐diving animals living in deep sea and stranded in shallow waters abnormally. Therefore they are assumed potentially unhealthy. Nevertheless, whale strandings could occur due to a number of reasons, such as confused navigation or distraction by human activities. Unfortunately, the potential stranding reasons for the three whales are unclear. Whatever, our results, especially from GM19, have a potential to be affected either by gastrointestinal disease or medical treatment and therefore should be used with caution when comparing with other individuals.

## CONCLUSIONS

In this study, we used high‐throughput sequencing technology to analyze microbial communities in the stomach, midgut, and hindgut of two stranded short‐finned pilot whales and in the hindgut of a stranded pygmy sperm whale. Our results indicated the markedly different microbial composition in the guts of the 2 stranded short‐finned pilot whales and hindgut of the stranded pygmy sperm whale. Interestingly, the most abundant OTUs were closely related to opportunistic and pathogenic strains, such as *B. fragilis* and *V. cidicii*, which can cause inflammatory bowel diseases. The results may provide a better understanding of the microbial world in the gastrointestinal tracts of different cetaceans and help us gain a better insight into the gut microbiomes of marine mammals. However, we have very limited information on the microorganisms found in marine mammals, and further studies of microbial communities in different marine mammal species from different marine environments should be conducted. In addition, metagenomics, transcriptomics, and proteomics should be used to understand functional information on gut microorganisms in marine mammals.

## CONFLICT OF INTEREST

No conflict of interest.

## Supporting information




**Figure S1** Phylogenetic tree based on the OTU1 (a), OTU2 (b), OTU9 (c), and OTU1247 (d) sequences that show the relationships between each OTU and related strains by using the maximum‐likelihood method with 1000 replications. OTU1 and OTU2 represent the most abundant OTUs in the gut samples from GM19. OTU9 and OTU1247 were shared in the samples of GM16 and GM19 and hindgut sample of KB. The scale bars indicate differences in the nucleotide sequences. GM16 refers to *Globicephala macrorhynchus* stranded in 2016; GM19 refers to *G. macrorhynchus* stranded in 2019; KB refers to *Kogia breviceps* stranded in 2014.
