# Supplementary figures and images for: Microbial diversity and structure in the gastrointestinal tracts of two stranded short‐finned pilot whales (Globicephala macrorhynchus) and a pygmy sperm whale (Kogia breviceps)
Source: Integr Zool. 2020 Dec 2;16(3):324–35. doi: 10.1111/1749-4877.12502 (PMC9292824; doi:10.1111/1749-4877.12502)

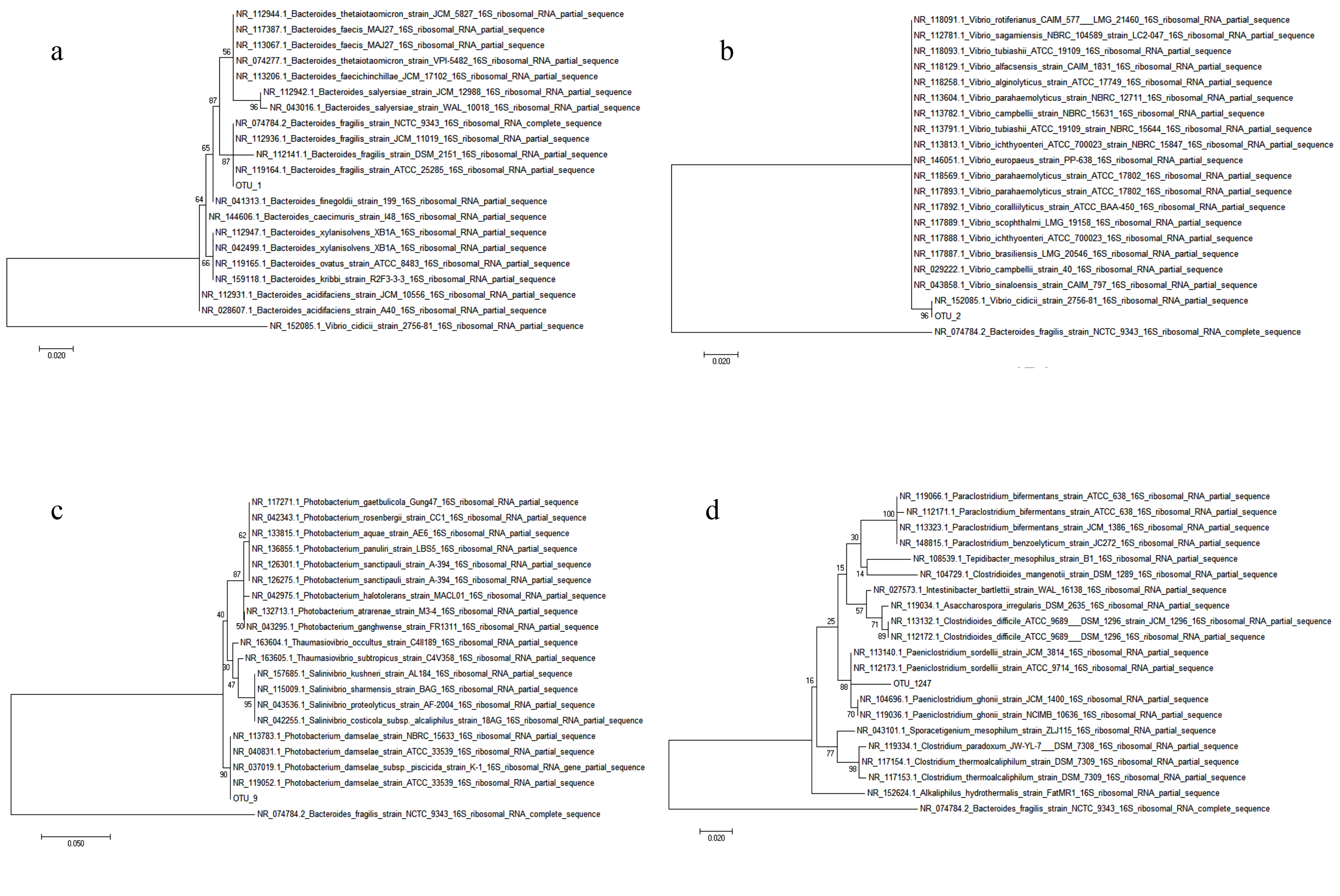

Supplement: Supplementary file 1 — Figure S1 Phylogenetic tree based on the OTU1 (a), OTU2 (b), OTU9 (c), and OTU1247 (d) sequences that show the relationships between each OTU and related strains by using the maximum‐likelihood method with 1000 replications. OTU1 and OTU2 represent the most abundant OTUs in the gut samples from GM19. OTU9 and OTU1247 were shared in the samples of GM16 and GM19 and hindgut sample of KB. The scale bars indicate differences in the nucleotide sequences. GM16 refers to Globicephala macrorhynchus stranded in 2016; GM19 refers to G. macrorhynchus stranded in 2019; KB refers to Kogia breviceps stranded in 2014. [file INZ2-16-324-s001.tif]
